# Determinants of prehospital coronary heart disease death

**DOI:** 10.1038/s41598-021-96575-2

**Published:** 2021-08-24

**Authors:** Ute Amann, Margit Heier, Christian Thilo, Jakob Linseisen, Christa Meisinger

**Affiliations:** 1 Independent Research Group Clinical Epidemiology, Helmholtz Zentrum München, German Research Center for Environmental Health (GmbH), Ingolstädter Landstraße 1, 85764 Neuherberg, Germany; 2 KORA Study Centre, University Hospital of Augsburg, Augsburg, Germany; 3 Institute of Epidemiology, Helmholtz Zentrum München, German Research Center for Environmental Health (GmbH), Neuherberg, Germany; 4 Department of Medical Clinic I—Cardiology, Hospital of Rosenheim, Rosenheim, Germany; 5 Chair of Epidemiology, University of Augsburg, At University Hospital Augsburg, Augsburg, Germany; 6 Institute for Medical Information Processing, Biometry, and Epidemiology—IBE, LMU Munich, Munich, Germany

**Keywords:** Cardiology, Epidemiology, Outcomes research, Risk factors

## Abstract

Limited data on prehospital and early in-hospital coronary heart disease (CHD) deaths is available. Aims of this study were to provide a comprehensive description on CHD cases and to analyse determinants of prehospital death. From a population-based myocardial infarction (MI) registry in Augsburg, Germany we included 12,572 CHD cases aged 25–74 years between 2003–2017 and 4754 CHD cases aged 75–84 years between 2009–2017. Multivariable logistic regression models were conducted to identify patient characteristics associated with prehospital death compared to 28-day survival. In patients aged 25–74 years, 1713 (13.6%) died prehospital, 941 (7.5%) died within the first 24 h in-hospital and 560 (4.5%) died within the 2nd and 28th day after the acute event; in patients aged 75–84 years the numbers were 1263 (26.6%), 749 (15.8%) and 329 (6.9%), respectively. In both age groups increasing age, actual smoking or nicotine abuse, previous MI, angina pectoris and previous stroke were more likely and hypertension was less likely in cases, who died prehospital compared to 28-day survivors. For example, in the 25–74 years old we revealed an adjusted odds ratio (OR) of 4.53 (95% CI 3.84–5.34) for angina pectoris and an OR of 0.69 (95% CI 0.57–0.85) for hypertension. In cases aged 25–74 years, an association of living alone (OR 1.26, 95% CI 1.06–1.49) and diabetes (OR 1.20, 95% CI 1.03–1.41) with prehospital death was found. Whereas in cases aged 75–84 years, chronic obstructive pulmonary disease (OR 2.20, 95%CI 1.69–0.2.85) was associated with prehospital death. In summary, we observed high prehospital and early in-hospital case fatality. Besides classical cardiac risk factors, the impact of living alone on prehospital death was more important in patients aged 25–74 years than in older patients.

## Introduction

Over the last decades, morbidity and mortality in acute myocardial infarction (MI) has declined in men and women of almost all European populations analysed^1^. The World Health Organization (WHO) multinational MONICA (MONItoring Trends and Determinants in CArdiovascular disease) project found that over the 10 years studied from the early 1980s, the fall in coronary heart disease (CHD) mortality rates in the population aged 35–64 years was two thirds determined by decline in coronary-event rates and one third by decline in case fatality^2^. Even among persons above 74 years the CHD event rates and the 28-day case fatality, defined as ‘as number of deaths within 28 days (mortality) divided by fatal plus non-fatal coronary events (morbidity) by 100’, has decreased in western countries such as Finland^3^ and Germany^4^. Besides this success, recent data of the population-based MI registry in Germany showed a prehospital case fatality of 27.8% in 25 to 84 years old men and 36.5% in women of the same age-range in 2016/2018. In addition, case fatality during the first 24 h after hospitalization was 11.6% in men and 12.9% in women^4^. Similarly, a high percentage of 28.9% with an out-of-hospital death due to CHD were reported from Sweden in a nationwide study during 1991 to 2006. The authors found that the relative contribution of out-of-hospital death to overall case fatality increased with time, especially among younger persons aged 35–54 years with 15.7% of all events dying outside the hospital and 1.9% dying in hospital within 28 days after hospital admission^5^.

Several causes were discussed to be responsible for an early death after an acute coronary event, ranging from age, comorbidities and cardiovascular (CV) risk factors of the individual person, to late diagnosis and late administration of reperfusion therapy due to delayed perception of typical MI symptoms and late call for emergency medical services or a delayed prehospital time in general^6^. In addition, some of the early death events are presumable sudden coronary deaths resulting from abrupt loss of heart function within minutes after the onset of symptoms in person with known or unknown CV disease^6,7^. As systematic assessment of prehospital case fatality in patients with CHD is complicated, less is known about the circumstances and characteristics of these patients^8^. To study determinants and correlates of early cardiac death due to a possible MI on a population level, epidemiological registries or surveys with additional information from death certificates and last treating physicians may provide useful information. In contrast to epidemiological MI registries, clinical or physician self-reported MI registries have the disadvantage of not collecting prehospital deaths and may under-register early in-hospital deaths^8^. In addition to a nearly complete case enrolment over long periods, epidemiological studies also provide the advantage to collect further information on patient characteristics and risk factors assessed with standardized questionnaires and procedures^9^. In case of national diagnosis and death registries, data linkage offers a valuable source for out-of-hospital CHD cases and their CV risk factors^5,10^. Several registry studies analysed trends in coronary event rates and CHD mortality^1–3,5^, but only few studies investigated patients’ characteristics associated with of out-of-hospital deaths^5,10–12^.

Aims of this study were firstly, to provide information on sociodemographic characteristics, medical history and CV risk factors among persons registered in a population-based MI registry from 2003 to 2017 by survival status with a special focus on prehospital deaths and early in-hospital deaths. Secondly, to analyse potential determinants associated with prehospital death compared to 28-day survival after the acute coronary event.

## Methods

### Study design and data source

The present observational study is based on data from the epidemiological MI registry in Augsburg, Germany, which was established in 1984 as part of the WHO MONICA Project. After the termination of MONICA in 1995, the Augsburg MI registry has been continued as part of KORA (Cooperative Health Research in the Region of Augsburg). Since 1984, population-based CHD event monitoring and acute coronary care recording is continuously done in the 25–74-year old population of the city of Augsburg and two surrounding counties (about 680,000 inhabitants). Besides the region’s major hospital, University Hospital of Augsburg, further seven hospitals participated in the MI registry. From 2009 onwards, the KORA MI registry was extended up to the 85-year old inhabitants of the study region. According to the MONICA manual^9^ registered events are classified depending on the length of survival into two main groups: (A) fatal cases and (B) non-fatal cases, and further into the following four subgroups: (A1) prehospital deaths, (A2) deaths within 24 h after hospitalization, (B1) cases surviving at least 24 h after hospitalization, but dying within 28 days after the acute event and (B2) cases surviving at least 28 days (28-day survivors). The methods of fatal and non-fatal case identification, diagnostic classification of events and data quality control in the KORA MI registry have been described in detail elsewhere^13,14^. Briefly, fatal CHD (ICD-9: 410–414) were identified using anonymised death certificates from the local health departments within the study region. Up to December 31, 2000, hospitalized cases were registered according to the diagnostic criteria of the WHO MONICA protocol^9^ using symptoms, electrocardiogram, enzyme such as creatine phosphokinase and necropsy evidence^14^. Since 2001, all patients with acute MI diagnosed according to the European Society of Cardiology and the American College of Cardiology have been registered^14,15^. Diagnostic evidence is used to classify the registered events as definite (with necropsy evidence) acute MI, possible acute MI, ischemic arrest, fatal cases with insufficient data, and non-events (‘false positives’)^13^. Validation of all CHD cases is performed by the physicians of the registry. For reasons of comparability, the epidemiologic MONICA algorithm has been applied in this study for the fatal CHD and non-fatal acute MI diagnosis for the whole study period. The documented troponin values that have been collected since 2001 were not considered for the epidemiologic acute MI diagnosis.

### Data collection

The procedure of data collection in the KORA MI registry depends on the two main registered groups as described above. For prehospital deaths and deaths within 24 h after hospitalization, data gathering was performed anonymously with the support of the local health departments and the participating hospitals in the study region through screening of death certificates in addition to a written standardized questionnaire, routinely sent by the local health department to the last treating physician. Non-fatal cases were interviewed shortly after intensive care and a review of medical charts were performed by trained study nurses using a standardized questionnaire. Both questionnaires (for fatal and non-fatal cases) collected sociodemographic characteristics, CV risk factors (e.g., hypertension, dyslipidaemia, diabetes mellitus, smoking or nicotine abuse), medical history of previous MI, stroke, chronic obstructive pulmonary disease (COPD), cancer, and information on the acute event reported either by patient, hospital chart or last treating physician. If the information on medical history from patient-report and medical chart differed, the chart information was used. Due to the different procedure of data collection in fatal and non-fatal cases, some patient characteristics such as obesity (reported by last treating physicians in fatal cases vs. defined according to the body weight and height measured during hospitalization) and smoking (nicotine abuse reported by last treating physicians vs. actual, former or never smoking reported by patients) were not identically or were totally missing such as information on cancer, and up to 2008 on COPD in non-fatal cases. Hospitalized patients who refused to participate were registered with information only on sex, age and date of the event.

### Ethics approval and consent to participate

Data collection of the KORA MI registry has been approved by the ethics committee of the Bavarian Medical Association (Bayerische Landesärztekammer) and the study was performed in accordance with the Declaration of Helsinki. All study participants with a non-fatal acute MI have given written informed consent. For fatal cases, data collection was performed anonymously as described above.

### Study population

**Figure 1:**
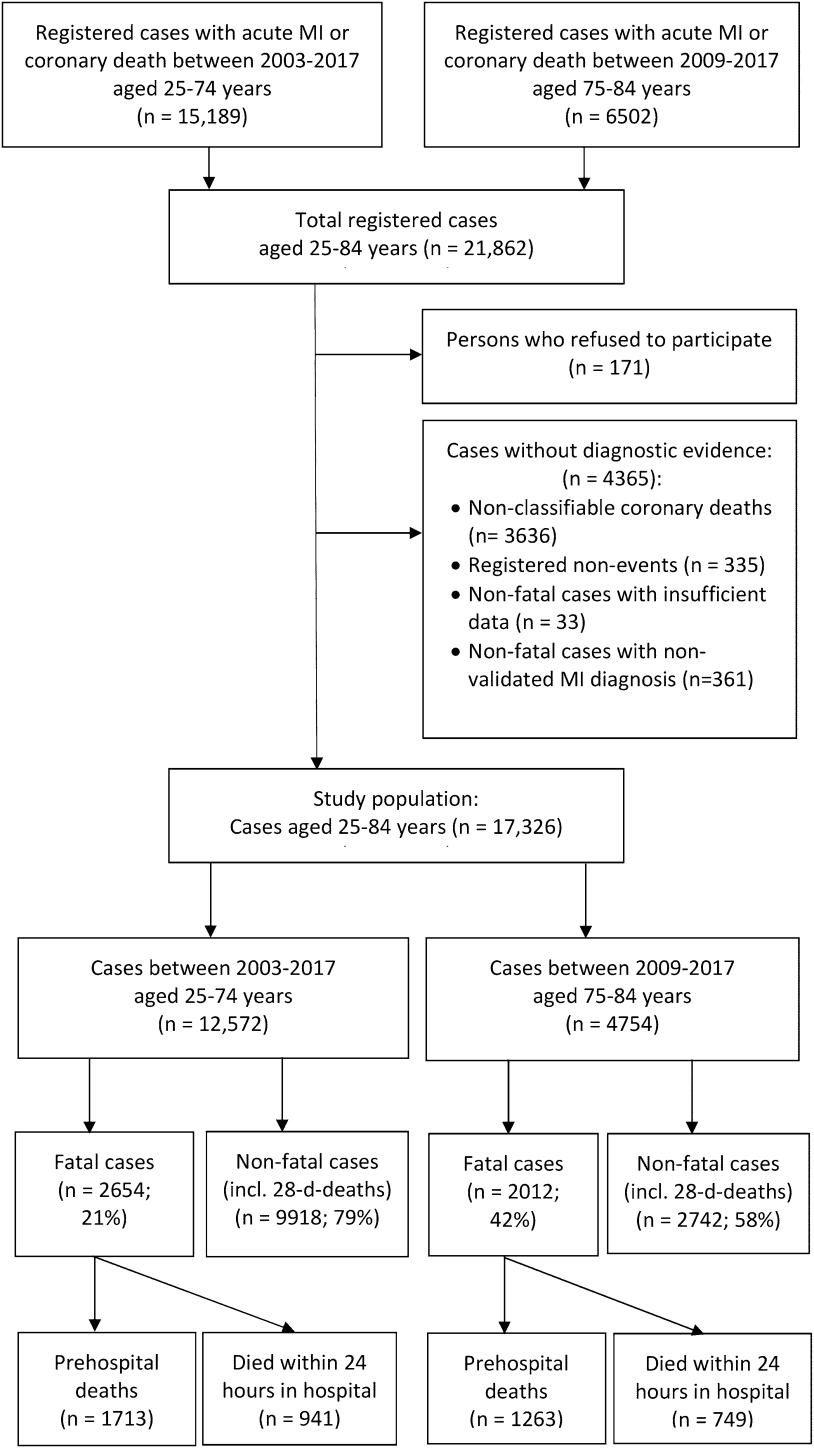
Study population (n = 17,326) from the KORA Myocardial infarction (MI) registry.

Out of the 21,862 persons aged 25–84 years who were consecutively enrolled in the KORA MI registry between January 1, 2003 (2009 for the 75–84-year old participants), and December, 31, 2017, we excluded 171 hospitalized persons who refused to participate and 4365 cases without diagnostic evidence (e.g., unclassifiable coronary death cases, registered non-events and non-fatal cases with no validated MI diagnosis). Thus, the study population of the present study covered 17,326 cases (12,572 cases aged 25–74 years between 2003 and 2017 and 4754 cases aged 75–84 years between 2009 and 2017) (Fig. 1).

### Statistical analysis and patient groups analysed

All analyses were performed separately for the 25–74-year old participants for the time period 2003–2017 and for the 75–84-year old participants from 2009 to 2017. To describe the characteristics of the cases categorical variables were expressed as absolute numbers and percentages (%) and continuous variables as median with interquartile range (25th and 75th percentiles). To analyse potential determinants associated with prehospital death (yes/no) multivariable logistic models were performed. As comparison group, the 28-day-survivors were used. The following variables were analysed by conducting a complete-case analysis: sex, age (continuous), living alone, previous MI, medical history of stroke, angina pectoris, hypertension, diabetes mellitus, dyslipidaemia, actual smoking/nicotine abuse (non-fatal/fatal cases), COPD (cases aged 75–84 years) and obesity reported by last treating physician (fatal cases) or defined as body mass index >  = 30 kg/m^2^ (non-fatal cases). These patients’ characteristics were cross tabulated with survival status (prehospital death vs. 28-day survival) using Chi^2^-test for categorical variables and the Kruskal–Wallis test (Wilcoxon Analysis) for continuous variables. As criterion for entry into the models, the explanatory variables—except for sex and age—had to meet the 0.2 significance level in the bivariate analysis. The final multivariable models included only factors which significantly (p < 0.05) contributed to the specific model using forward selection technique. Due to missing values, we excluded 854 prehospital death cases and 940 cases of 28-day survivors of the 25–74-year old participants in the final model, and n = 359 prehospital death cases and 409 cases of 28-day survivors of the 75–84-year old participants, respectively. As sub-analyses, the regression models were recalculated using all short-term fatal cases (prehospital deaths and deaths within the first 24 h after hospitalization) compared to 28-day survivors.

All analyses were performed using SAS version 9.4 (SAS Institute Inc., Cary, North Carolina).

## Results

### Sample characteristics by survival status

From the 17,326 CHD cases included in this study, 12,660 were non-fatal and 4666 fatal cases. The distribution between non-fatal and fatal cases was 79:21 in the 25–74 years old during the time period 2003 and 2017, and 58:42 in the 75–84 years old persons during 2009 and 2017. In patients aged 25–74 years, 1713 (13.6%) died prehospital (PHD), 941 (7.5%) died within the first 24 h after hospitalization (24hD) and 560 (4.5%) died within the 2nd and 28th day after the acute event (28dD); in patients aged 75–84 years the numbers were 1263 (26.6%), 749 (15.8%), and 329 (6.9%), respectively (Fig. 1 and Table 1).

**Table 1:** Characteristics of the study population (n = 17,326) by age-group and the epidemiologic MONICA algorithm for the fatal coronary heart disease and non-fatal acute MI diagnosis.

	25–74 years old cases between 2003–2017 (n = 12,572)				75–84 years old cases between 2009–2017 (n = 4754)			
	Fatal (n = 2654)		Non-fatal (n = 9918)		Fatal (n = 2012)		Non-fatal (n = 2742)	
	PHD	24hD	28dD	28dS	PHD	24hD	28dD	28dS
Included cases, n	1713	941	560	9358	1263	749	329	2413
**Socio-demographics**								
Mean (SD) age, y	64.7 (8.1)	66.1 (7.6)	65.6 (7.5)	61.0 (9.5)	80.1 (2.8)	79.7 (2.9)	79.9 (2.8)	79.1 (2.8)
Median (IQR) age, y	67 (10)	68 (9)	67 (10)	63 (15)	81 (5)	80 (5)	80 (4)	79 (4)
Women, n (%)	380 (22.2)	214 (22.7)	148 (26.4)	2259 (22.1)	516 (40.9)	283 (37.8)	141 (42.9)	1010 (41.9)
**Nation, n (%)**								
German	1404 (82.0)	730 (77.6)	512 (91.4)	8372 (89.5)	1090 (86.3)	564 (75.3)	311 (94.5)	2288 (94.8)
Other	77 (4.5)	50 (5.3)	44 (7.9)	980 (10.5)	27 (2.1)	18 (2.4)	18 (5.5)	116 (4.8)
Missing information	232 (13.5)	161 (17.1)	4 (0.7)	6 (0.1)	146 (11.6)	167 (22.3)	0	9 (0.4)
**Living alone, n (%)**								
Yes	309 (18.0)	54 (5.7)	44 (7.9)	1755 (18.8)	307 (24.3)	99 (13.2)	47 (14.3)	618 (25.6)
Missing information	556 (32.5)	436 (46.3)	166 (29.6)	465 (5.0)	176 (13.9)	274 (36.6)	77 (23.4)	156 (6.5)
**Obesity, n (%)**								
Yes	329 (19.2)	112 (11.9)	82 (14.6)	2592 (27.7)	188 (14.9)	71 (9.5)	34 (10.3)	508 (21.1)
Missing information	613 (35.8)	368 (39.1)	304 (54.3)	375 (4.0)	186 (14.7)	201 (26.8)	152 (46.2)	139 (5.8)
**Nicotine abuse, n (%)**								
Yes	405 (23.6)	196 (20.8)	n.a	n.a	132 (10.5)	96 (12.8)	n.a	n.a
Missing information	605 (35.3)	363 (38.6)	n.a	n.a	185 (14.7)	203 (27.1)	n.a	n.a
**Smoking**								
Actual	n.a	n.a	148 (26.4)	3425 (36.6)	n.a	n.a	25 (7.6)	157 (6.5)
Former	n.a	n.a	118 (21.1)	2944 (31.5)	n.a	n.a	53 (16.1)	808 (33.5)
Never	n.a	n.a	47 (8.4)	2416 (25.8)	n.a	n.a	52 (15.8)	1122 (46.5)
Missing information	n.a	n.a	247 (44.1)	573 (6.1)	n.a	n.a	199 (60.5)	326 (13.5)
**Medical history, n (%)**								
**Hypertension**								
Yes	1185 (69.2)	601 (63.9)	413 (73.8)	7264 (77.6)	985 (78.0)	511 (68.2)	267 (81.2)	2172 (90.0)
Missing information	235 (13.7)	180 (19.1)	3 (0.5)	0	135 (10.7)	165 (22.0)	0	0
**Diabetes mellitus**								
Yes	625 (36.5)	360 (38.3)	252 (45.0)	3015 (32.2)	505 (40.0)	272 (36.3)	145 (44.1)	992 (41.1)
Missing information	249 (14.5)	180 (19.1)	2 (0.4)	1 (0.01)	144 (11.4)	166 (22.2)	0	1 (0.04)
**Dyslipidaemia**								
Yes	857 (50.0)	367 (39.0)	226 (40.4)	5894 (63.0)	641 (50.8)	306 (40.9)	145 (44.1)	1344 (55.7)
Missing information	293 (17.1)	205 (21.8)	4 (0.7)	3 (0.03)	173 (13.7)	194 (25.9)	0	0
**Previous MI**								
Yes	716 (41.8)	433 (46.0)	148 (26.4)	1699 (18.2)	607 (48.1)	422 (56.3)	78 (23.7)	594 (24.6)
Missing information	233 (13.6)	146 (15.5)	0	4 (0.04)	159 (12.6)	110 (14.7)	0	0
**Angina pectoris**								
Yes	728 (42.5)	309 (32.8)	109 (19.5)	1569 (16.8)	510 (40.4)	228 (30.4)	55 (16.7)	499 (20.7)
Missing information	442 (25.8)	311 (33.1)	25 (4.5)	46 (0.5)	291 (23.0)	298 (39.8)	7 (2.1)	11 (0.5)
**Stroke**								
Yes	229 (13.4)	109 (11.6)	81 (14.5)	656 (7.0)	313 (24.8)	138 (18.4)	58 (17.6)	364 (15.1)
Missing information	298 (17.4)	206 (21.9)	85 (15.2)	270 (2.9)	167 (13.2)	181 (24.2)	26 (7.9)	67 (2.8)
**Cancer**								
Yes	123 (7.2)	99 (10.5)	n.a	n.a	182 (14.4)	136 (18.2)	n.a	n.a
Missing information	633 (37.0)	373 (39.6)	n.a	n.a	245 (19.4)	219 (29.2)	n.a	n.a
**COPD**								
Yes	239 (14.0)	112 (11.9)	48 (8.6)	323 (3.5)	195 (15.4)	99 (13.2)	36 (10.9)	221 (9.2)
Missing information	612 (35.7)	362 (38.5)	255 (45.5)	3798 (40.6)	179 (14.2)	184 (24.6)	1 (0.3)	4 (0.2)

*PHD* pre-hospital deaths, *24hD* death within the first 24 h after hospitalization, *28dD* death within 2nd and 28th day after acute event, *28dS* 28-day survivors, *n.a.* not available due to differences in data source and data collection regarding fatal and non-fatal cases, *COPD* chronic obstructive pulmonary disease.

Table 1 shows the sociodemographic characteristics and the medical history for each of the four subgroups of the two age groups and time periods. Due to different data sources for fatal and non-fatal cases used in our registry, some information was not available as described in the method part. In addition, we observed higher missing values in PHD cases compared to 28-day survivors. In PHD cases, nicotine abuse was known in 23.6% of the 25–74 years old and in 10.5% of the 75–84 years old. Among 28-day survivors, actual smoking was reported by 36.6% of the 25–74 years old and by 6.5% of the older age-group. Angina pectoris and previous MI was more often seen in fatal compared to non-fatal cases. For example, in the 75–84 years old, previous MI was known in 48.1% of PHD and 56.3% of 24hD cases versus 23.7% of 28dD and 24.6% of the 28-day survivors.

Table 2 provides additional characteristics on witness and place of death, autopsy and length of survival for fatal cases. The frequencies observed were similar in both age groups except for autopsy (e.g., ‘no autopsy performed' in PHD: 95.2% of 25–74 years old versus 99.4% of 75–84 years old cases) and length of survival after the acute event. Despite differences in missing frequencies for the variable ‘time between pain and death’, we revealed a considerable number of patients dying within one hour after pain onset. In PHD and 24hD cases, the frequencies of cardiac death within 1 h after pain onset were 38.5% in 25–74 years old and 24.4% in 75–84 years old cases, and 18.3% in 25–74 years old and 12.3% in 75–84 years old cases, respectively (Table 2).

**Table 2:** Additional characteristics of the fatal cases (n = 4666).

	25–74 years old fatal cases (n = 2654)		75–84 years old fatal cases (n = 2012)	
	PHD	24hD	PHD	24hD
Included cases, n	1713	941	1263	749
**Witness of death, n (%)**				
Physician	90 (5.3)	744 (79.1)	71 (5.6)	517 (69.0)
Other witness	829 (48.4)	106 (11.3)	597 (47.3)	120 (16.0)
No witness	612 (35.7)	23 (2.4)	459 (36.3)	36 (4.8)
Missing information	182 (10.6)	68 (7.2)	136 (10.8)	76 (10.2)
**Autopsy, n (%)**				
Yes (definite MI, in case of ICD-9 = 410, 411 or 412)	54 (3.2)	55 (5.8)	6 (0.5)	12 (1.6)
Yes (possible MI, in case of ICD-9 = 413 or 414)	14 (0.8)	14 (1.5)	2 (0.2)	2 (0.3)
No autopsy performed	1631 (95.2)	849 (90.2)	1255 (99.4)	727 (97.1)
Missing information	14 (0.8)	23 (2.4)	0	8 (1.1)
**Place of death, n (%)**				
At home, at work or on the road	1541 (90.0)	0	1138 (90.1)	0
At transport to hospital	12 (0.7)	0	2 (0.2)	0
At hospital admission	0	135 (14.4)	n.a	67 (9.0)
At intensive care	0	515 (54.7)	n.a	298 (39.8)
At hospital, rehabilitation or hospice care	60 (3.5)	291 (30.9)	83 (6.6)	384 (51.3)
Others or insufficient data	100 (5.8)	0	40 (3.2)	0
**Time between pain and death, n (%)**				
1 h or less	660 (38.5)	172 (18.3)	308 (24.4)	92 (12.3)
1–24 h	206 (12.0)	250 (26.6)	128 (10.1)	147 (19.6)
Mostly likely < = 24 h	196 (11.4)	51 (5.4)	154 (12.2)	55 (7.3)
> 24 h	80 (4.7)	199 (21.2)	91 (7.2)	178 (23.8)
Insufficient data	571 (33.3)	269 (28.6)	582 (46.1)	277 (37.0)

*PHD* pre-hospital deaths, *24hD* deaths within the first 24 h after hospitalization, *MI* myocardial infarction, *ICD-9* International Classification of Diseases, 9th revision.

### Determinants associated with prehospital death

Results of the multivariable logistic regression models are provided in Table 3. In both age groups increasing age, actual smoking or nicotine abuse, previous MI, angina pectoris and previous stroke were more likely and hypertension was less likely in cases with prehospital death compared to 28-day survivors. For example, in the 25–74 years old we revealed an odds ratio (OR) of 4.53 (95% CI 3.84–5.34) for angina pectoris and an OR of 0.69 (95% CI 0.57–0.85) for hypertension. An association of living alone (OR 1.26, 95% CI 1.06–1.49) and diabetes (OR 1.20, 95% CI 1.03–1.41) with prehospital death was only found in cases aged 25–74 years. In cases aged 75–84 years, COPD (OR 2.20, 95%CI 1.69–2.85) was associated with prehospital death.

**Table 3:** Factors associated with prehospital death compared to 28-day survival.

	OR [95% CI]	p Value
**Model 1: Cases aged 25–74 years, 2003–2017 (n = 9277)**^a^		
Age (cont.)	1.05 [1.04–1.06]	< 0.0001
Living alone (yes vs. no)	1.26 [1.06–1.49]	0.01
Actual smoking/nicotine abuse (yes vs. no)	1.47 [1.24–1.75]	< 0.0001
Hypertension (yes vs. no)	0.69 [0.57–0.85]	0.0003
Diabetes mellitus (yes vs. no)	1.20 [1.03–1.41]	0.02
Dyslipidaemia (yes vs. no)	0.69 [0.59–0.82]	< 0.0001
Previous MI (yes vs. no)	1.87 [1.58–2.22]	< 0.0001
Angina pectoris (yes vs. no)	4.53 [3.84–5.34]	< 0.0001
Previous stroke (yes vs. no)	2.09 [1.68–2.61]	< 0.0001
**Model 2: Cases aged 75–84 years, 2009–2017 (n = 2908)**^b^		
Age (cont.)	1.17 [1.14–1.21]	< 0.0001
Obesity^c^ (yes vs. no)	0.80 [0.64–0.99]	0.04
Actual smoking/nicotine abuse (yes vs. no)	1.65 [1.21–2.23]	0.001
Hypertension (yes vs. no)	0.53 [0.40–0.71]	< 0.0001
Previous MI (yes vs. no)	2.28 [1.90–2.73]	< 0.0001
Angina pectoris (yes vs. no)	3.21 [2.68–3.86]	< 0.0001
Previous stroke (yes vs. no)	2.03 [1.64–2.51]	< 0.0001
COPD (yes vs. no)	2.20 [1.69–2.85]	< 0.0001

*MI* myocardial infarction, *COPD* chronic obstructive pulmonary disease.

^a^1794 observations (n = 854 prehospital death cases; n = 940 cases of 28-day survivors) were deleted due to missing values for the explanatory variables. COPD was not included in this model as information was not available up to 2008.

^b^Note: 768 observations (n = 359 prehospital death cases; n = 409 cases of 28-day survivors) were deleted due to missing values for the explanatory variables.

^c^Obesity reported by last treating physicians (fatal cases) or defined as body mass index >  = 30 kg/m^2^ (non-fatal cases).

The sub-analyses including all short-term fatal cases (PHD and 24hD) revealed results similar to those in Table 3, with one exception; in 25–74 years old cases, the variable ‘living alone’ was not found to be associated with the combined outcome of prehospital and early in-hospital death (data shown in Supplementary Table 1).

## Discussion

We performed an analysis of prehospital and early in-hospital CHD deaths and investigated patients’ characteristics associated with prehospital death in comparison with at least 28-day survival based on data of 17,326 participants from the epidemiological KORA MI registry in Augsburg, Germany. We found that 13.6% of the included cases aged 25–74 years and 26.6% of included cases aged 75–84 years died before reaching a hospital. In addition, 7.5% of the 25–74 years old and 15.8% of the 75–84 years old patients died within the first 24 h after hospitalization. Prehospital death after the acute event was associated with increasing age, nicotine abuse or actual smoking, previous MI, angina pectoris, previous stroke and absence of hypertension in both age groups. In the 25–74 years old cases, living alone, diabetes mellitus and absence of hyperlipidaemia were also found to be associated with prehospital death. In the elderly group where information on COPD was available during the total study period, persons with COPD, and non-obese persons more likely died before reaching a hospital.

Our findings of a high prehospital and early in-hospital case fatality are in concordance with prior studies^1,3,5^. For example, Koukkunen et al.^3^ examined 30,561 suspected acute MIs in persons aged 75–99 years in Finland and found an age-standardized prehospital case fatality among persons aged 75–84 years of 25.5% in men and 20.2% in women, that is similar to our prehospital case fatality of 26.6% among the 75–84 years old patients. Also, a nationwide Swedish study including 384,597 cases aged 35 to 84 years with a first major coronary event occurring between 1991 and 2006 found a high percentage of 28.9% out-of-hospital deaths and further 9.5% who died in the hospital or within 28 days after hospitalization^5^.

The high frequencies of about 39% in the 25–74 years old and 24% in the 75–84 years old cases observed in our analysis regarding length of survival after pain onset of less than one hour indicates that sudden coronary death (SCD) might be the main cause of death in these patients. This assumption is encouraged by the small proportion of present physicians (5–6%) and the high frequency of 36% among prehospital fatal cases with no witness for testifying the out-of-hospital deaths. A prospective study in France estimated that 35% out-of-hospital sudden deaths were due to acute MI^16^. The Finnish Genetic Study of Arrhythmic Events^17^ found an even higher proportion of sudden deaths in relation to CHD. Vähätalo et al.^17^ reported that in 74.8% out of 5869 individuals with autopsy-verified SCD, the cause of sudden death was a coronary artery disease; however, in 1322 individuals (42.4%) with SCD a silent MI was detected although no prior diagnosis of a coronary artery disease was recorded. As systematic assessment of SCD is complicated, the yearly rate varied widely between 18.6 and 128 cases per 100,000 inhabitants among different countries. In a German district in Lower Saxony with 190,000 inhabitants, the incidence of sudden cardiac death was found to be stable between 2002 and 2009, and was estimated to be 81 cases per 100,000 inhabitants per year^7^. As shown with our data, an epidemiological MI registry could provide regular data on SCD rate within a defined population of about 680,000 inhabitants.

Previously, it was reported, that 62% of young persons with SCD experienced angina pectoris before death and 46% had contacts with the health care system because of cardiac symptoms^18^. We found that established risk factors of MI such as increasing age, angina pectoris, previous MI, previous stroke, smoking and diabetes mellitus were more likely in prehospital fatal cases compared to 28-day survivors. However, we observed lower rates of hypertension in fatal cases, possible due to underdiagnosis or missing information. In accordance with a previous study^5^, we found that hypertension was inversely associated with prehospital death in both age groups. This may be a result of underdiagnosed hypertension as mentioned above as well as potential treatment with antihypertensive drugs and therefore better control of blood pressure in patients with known hypertension. Drug prescribing could also be one reason why cases aged 25–74 years who had known dyslipidaemia were less likely to die out-of-hospital. From modelling studies in CHD, it was demonstrated that primary prevention and control of risk factors are more important for a decrease in case fatality than interventions^5^. Importantly, Dudas et al.^5^ found an increasing temporal trend during 1991 to 2006 in out-of-hospital death with each successive calendar year, in cases with a first major coronary event.

In accordance with prior studies, our analyses showed that CV risk factors^10^, living alone^11,12^, but not sex^11^, was associated with prehospital death. The significance of living alone disappeared in our sub-analyses within the 25–74 aged cases with prehospital or early in-hospital death, strengthening the impact of living alone on prehospital death. In addition, Sorlie et al.^11^ found in a sample of the US population between 1979 and 1989 a higher OR of out-of-hospital death due to CHD (OR 1.44, 95% CI 1.34–1.55) than in all cause of death (OR 1.60, 95% CI 1.49–1.71) among persons living alone compared with a household size of more than one person. The authors discussed, that in case of a heart attack a person may be either unable to call for emergency help due to severe chest pain, or a spouse in the household may lead to earlier recognition of the cardiac warning symptoms and therefore may decide to notify emergency services rapidly. The observed absence of a medical witness or any other witness of death in our prehospital death cases supports the hypothesis that living alone is a relevant predictor of prehospital cardiac death, especially in persons below 75 years.

Our study adds to the hypothesis that obesity may be a protective factor for prehospital death in persons with acute coronary event 75 years and older. Importantly, when interpreting this result, one should keep in mind the limited information available on obesity especially regarding category or stages of obesity in fatal cases, where we received the information from the last treating physician. Therefore, misclassification cannot be ruled out. The findings could also be due to the known ‘obesity paradox’ observed in epidemiological studies defined as unexpected results that obese people seem to live longer than their normal- or underweight counterparts with CV disease^19^. However, geriatric studies reported that obesity was positive related with survival and health status and the cut-off point of 25 kg/m^2^ for overweight—classified according to the WHO guidelines—may be overly restrictive in adults aged 64 years or older^19,20^. Neeland et al.^20^ found even a U-shaped association between body mass index category and 3-year all-cause mortality after ST-segment elevation MI in patients aged >  = 65 years surviving to hospital discharge; where, patients with mild obesity (30–34.9 kg/m^2^) were at lowest risk. Finally, as long as the protective effects of adiposity in aged people with CV disease is not known, our results need to be considered carefully and will need further confirmation.

### Strength and limitations

Major strength of the present study is the setting of a population-based MI registry including cases of a clinically confirmed MI and validated coronary death consecutively registered in a defined study region including urban and rural areas. Furthermore, our research covers recent data up to 2017, and included a number of additional characteristics of fatal cases such as length of survival, witness of death, medical history and nicotine abuse that were not incorporated in previous investigations. In addition, we were able to report data on sudden and non-sudden coronary deaths. From 2009 onwards, our MI registry was extended to elderly persons up to 84 years old; therefore, additional analyses for an increasing patient population with CHD could be performed.

Some limitations for interpretation of our findings should be noticed. Firstly, our data on fatal cases contains a considerable number of missing values that might have caused information bias due to potential underdiagnosis of medical conditions or risk factor presence, leading to possible over- or underestimation of reported odds ratios associated with prehospital death. Altogether, the high percentage of missing information on variables studied may have also caused selection bias through misclassification regarding our studied groups. Secondly, several changes in emergency medical services, care and treatment of acute MI occurred during our study period between 2003 to 2017 that possibly influenced survival after an acute coronary event. Subsequently, the composition of our defined groups might converge each other over time with the potential to attenuate the reported odds ratios. A further limitation is that information on life expectancies and health status as well as on biomarkers and genetic susceptibility of the individuals was not available and may has determined the underlying mechanism for early coronary death versus 28-day survival in our sample. Finally, the autopsy rate is very low in our data; therefore, misclassification cannot be ruled out and may have caused overestimation of CHD deaths.

## Conclusions

In summary, we observed a high prehospital and early in-hospital case fatality after an acute coronary event and revealed that a considerable number of patients died of sudden cardiac death within one hour after pain onset. We found that established CV risk factors such as increasing age, angina pectoris, previous MI, previous stroke, smoking and diabetes mellitus were more likely in prehospital fatal cases compared to 28-day survivors. Moreover, the impact of living alone on out-of-hospital cardiac death seems particularly important in persons aged 25–74 years. To achieve a further decrease in CHD case fatality, primary prevention with control of risk factors as well as public awareness of heart attack symptoms are still major challenges for the society.

## Supplementary Information

Supplementary Information.

## Data Availability

The data will not be shared. Due to restrictions from Helmholtz Zentrum München, data are available upon request for any researcher based on a standard agreement on data provision within the KORA Research Platform.
