# Supplementary material for: Determinants of prehospital coronary heart disease death
Source: Sci Rep. 2021 Aug 24;11:17134. doi: 10.1038/s41598-021-96575-2 (PMC8385003; doi:10.1038/s41598-021-96575-2)
Supplement: Supplementary file 1 — Supplementary Information. [file 41598_2021_96575_MOESM1_ESM.docx]

**Supplementary information of the manuscript with the title:**

**Determinants of prehospital death in patients with acute coronary heart disease**

|  | **OR [95% CI]** | **p Value** |
| --- | --- | --- |
| **Model 1: Cases aged 25-74 years, 2003-2017 (n=9469)^a^** |  |  |
| Age (cont.) | 1.06 [1.05-1.07] | <.0001 |
| Actual smoking/nicotine abuse (yes vs. no) | 1.42 [1.23-1.65] | <.0001 |
| Hypertension (yes vs. no) | 0.68 [0.57-0.81] | <.0001 |
| Diabetes mellitus (yes vs. no) | 1.30 [1.13-1.49] | 0.0002 |
| Dyslipidaemia (yes vs. no) | 0.56 [0.49-0.65] | <.0001 |
| Previous MI (yes vs. no) | 2.19 [1.89-2.54] | <.0001 |
| Angina pectoris (yes vs. no) | 4.17 [3.62-4.82] | <.0001 |
| Previous stroke (yes vs. no) | 2.02 [1.66-2.46] | <.0001 |
| **Model 2: Cases aged 75-84 years, 2009-2017 (n=3313)^b^** |  |  |
| Age (cont.) | 1.15 [1.12-1.18] | <.0001 |
| Obesity^c^ (yes vs. no) | 0.69 [0.56-0.84] | 0.0003 |
| Actual smoking/nicotine abuse (yes vs. no) | 1.79 [1.37-2.34] | <.0001 |
| Hypertension (yes vs. no) | 0.57 [0.44-0.73] | <.0001 |
| Previous MI (yes vs. no) | 2.68 [2.28-3.15] | <.0001 |
| Angina pectoris (yes vs. no) | 3.09 [2.62-3.64] | <.0001 |
| Previous stroke (yes vs. no) | 1.79 [1.47-2.17] | <.0001 |
| COPD (yes vs. no) | 1.94 [1.53-2.46] | <.0001 |

**Supplementary Table 1.** Factor associated with either prehospital death or death within the first 24 hours after hospitalization (early death cases) compared to 28-day survival. *MI,* myocardial infarction; *COPD,* chronic obstructive pulmonary disease.

^a^ Note: 2543 observations (n=1430 early death cases; n=1113 cases of 28-day survivors) were deleted due to missing values for the explanatory variables. COPD was not included in this model as information was not available up to 2008.

^b^ Note: 1112 observations (n=703 early death cases; n=409 cases of 28-day survivors) were deleted due to missing values for the explanatory variables.

^c^ Obesity reported by last treating physicians (fatal cases) or defined as body mass index >=30 kg/m² (non-fatal cases).
